# Factors Associated with Quality of Life among People with Atrial Fibrillation: Jordan Atrial Fibrillation Registry Study

**DOI:** 10.3390/medicina60081262

**Published:** 2024-08-04

**Authors:** Ahmad M. Al-Bashaireh, Osama Alkouri, Abdulhafith Alharbi, Yousef Khader, Ayman Hammoudeh, Yousef Aljawarneh, Nader E. Alotaibi, Omar Qaladi, Anas Ababneh, Tim Schultz

**Affiliations:** 1 Department of Nursing, Faculty of Health Sciences, Higher Colleges of Technology, Abu Dhabi P.O. Box 1626, United Arab Emirates; aalbashaireh@hct.ac.ae (A.M.A.-B.); yaljawarneh@hct.ac.ae (Y.A.); 2 Faculty of Nursing, Yarmouk Univerity, P.O. Box 566, Irbid 21163, Jordan; anas.ababneh@yu.edu.jo; 3 College of Nursing, University of Hail, Hail 21424, Saudi Arabia; ay.alharbi@uoh.edu.sa; 4 Department of Public Health, Community Medicine, Jordan University of Science and Technology, Irbid 21163, Jordan; yskhader@just.edu.jo; 5 Istishari Hospital, Amman 840431, Jordan; a.hammoudeh@istisharihospital.com; 6 Medical Surgical Nursing Department, College of Nursing, King Saud University, Riyadh 12372, Saudi Arabia; alnader@ksu.edu.sa; 7 Community and Psychiatric Mental Health Nursing Department, King Saud University, Riyadh 11362, Saudi Arabia; oqaladi@ksu.edu.sa; 8 Flinders Health and Medical Research Institute, Flinders University, Sturt Road, Bedford Park South Australia 5042, P.O. Box 2100, Adelaide, SA 5001, Australia; timothy.schultz@flinders.edu.au

**Keywords:** atrial fibrillation, quality of life, AFEQT, Jordan

## Abstract

*Background and Objectives:* Atrial fibrillation (AF) is a common arrhythmia that may adversely affect the quality of life (QoL). Several factors could be associated with the QoL among patients with AF; however, evidence regarding these factors is still limited and controversial. Therefore, this study aimed to identify the level of QoL and its associated factors among Jordanian patients with AF. *Subjects and methods:* A case study design was implemented. A sample of 620 participants were recruited from 28 outpatient clinics registered in the Jordan atrial fibrillation registry AF (JoFIB). Data on QoL were gathered through the self-reported Atrial Fibrillation Effect on Quality-of-life tool (AFEQT). A QoL questionnaire was validated in this population before starting this study. The cardiac nurse then provided the research assistant with data relating to patients’ characteristics and associated comorbidities. *Results:* The overall AFEQT scores were positively skewed (median 21.3, IQR: 14.4–31.9). This pattern was reflected for the AFEQT sub-scales ‘Symptoms’ (20.8, 8.3–33.3), ‘Daily activities’ (16.7, 10.4–27.1), and ‘Treatment concerns’ (27.8, 19.4–41.7), whereas ‘Treatment satisfaction’ was negatively skewed (91.7, 83.3–91.7). Patients in the higher quartiles, indicating a better QoL, tended to be younger and were less likely to experience dyslipidemia, stroke, pulmonary hypertension, or other comorbidities. Over 90% of patients were currently experiencing AF, and patients with a better QoL tended to be less likely to be currently experiencing AF and more likely to have had their latest episode of AF more than a month ago (compared to less than a month ago). Age, BMI, dyslipidemia, heart failure, COPD, CAD, history of ablation, and the use of anticoagulants were significantly associated with the overall AFEQT score (R^2^ = 0.278). *Conclusions:* This study demonstrates that AF Jordanian patients had low levels of QoL. Patients in higher quartiles for the overall AFEQT score were younger, with fewer disease comorbidities and less experience of current AF episodes. Several modifiable and non-modifiable factors were associated with QoL in AF patients. Age, BMI, dyslipidemia, heart failure, COPD, CAD, history of ablation, and the use of oral anticoagulants were significantly associated with the overall AFEQT score. Healthcare providers should target these factors as indicators or interventions for which QoL is continuously monitored.

## 1. Introduction

Atrial fibrillation (AF) is the most common tachyarrhythmia, with a prevalence of 3.2% in adults, and it is characterized by inefficient atrial contraction due to uncoordinated electrical impulses [1]. The impact of AF varies significantly at the national and regional levels [2,3]. Global increases have been observed in occurrences, fatalities, and disability-adjusted life years (DALYs) between 1990 and 2019. Overall, 33.31 million cases of AF, 219.4 thousand deaths, and 65.80 million DALYs were reported worldwide in 2019 [3]. Due to the increased prevalence of detection rates and risk factors, the number of AF cases is anticipated to double by 2060 [4,5].

AF can significantly impact patients’ quality of life (QoL) for various reasons, including the debilitating symptoms of AF such as chest pain, fatigue, and dyspnea, the adverse effects related to AF medication, frequent hospital admissions [6,7,8], and health-related severe consequences including heart failure, stroke, cognitive decline, thromboembolism, and death [5,8,9,10]. Additionally, AF care and its potential complications are expensive, making up about 2% of healthcare costs in high-income countries [4].

QoL in patients with AF can be influenced by various factors, including sociodemographic characteristics [10], clinical characteristics [6,11], and psychological factors [12,13]. For example, one study found that females and younger patients had a poorer QoL among patients with AF [10]. A recent study of 1097 old patients with AF found that QoL negatively impacted women, patients with depression, low social support, and fair-to-poor self-rated health, and those with chronic associated comorbidities [13].

Enhancing QoL and identifying its associated factors is the primary goal of AF management. This helps alleviate patients’ discomfort, deliver patient-centered care, and achieve favorable clinical and functional outcomes [13]. However, the evidence regarding the factors associated with QoL among patients with AF is still limited and controversial [13,14,15]. Understanding the QoL in AF patients within the Jordanian context is crucial for several reasons. Jordan’s healthcare system includes a mix of public, private, and military sectors, with disparities in access and quality of care [16,17]. Jordan’s geopolitical situation, including its proximity to conflict zones, can contribute to heightened stress and anxiety among its population. The influx of refugees also strains the healthcare system, potentially affecting the availability and quality of care for all patients, including those with AF [16,17]. Moreover, cultural factors, such as food habits, lifestyles, and social support systems, differ greatly from those in Western countries, potentially influencing the management and consequences of AF [18]. The healthcare infrastructure, fiscal restrictions, and regional health policy all influence patient experiences and outcomes [17]. Furthermore, no studies targeting QoL and its associated factors were conducted in Jordan. Therefore, this study aimed to assess the level of QoL and its associated factors among Jordanian patients with AF.

## 2. Methods

### 2.1. Design and Sampling

A case study was conducted on a sample of 620 Jordanian patients with AF attending outpatient cardiac clinics. The patients were recruited from 28 healthcare settings registered in the Jordan atrial fibrillation registry AF (JoFIB). During follow-up visits, patients were selected if their age was 18 years or more and having a primary diagnosis of AF. In this study, patients were excluded if they failed to complete the survey, were incapable of providing signed informed consent, had a history of mental illness, or had cognitive disabilities. Participants who were illiterate or needed help understanding the Arabic language were only eligible to participate in this study if they had a friend or companion who was able to help them.

### 2.2. Sample Size Estimation

The G*Power 3.1.9.4 was used to calculate the sample size. A linear multiple regression model with the following parameters: R^2^ = 0.08 at a power of 80% with 20 independent variables, and α = 0.05 requires 279 subjects. This study successfully recruited 620 subjects.

### 2.3. Ethical Considerations

The current study adheres to the ethical standards defined in the Helsinki Declaration of 1975, as revised in 2008, and the relevant institutional and national guidelines on human studies. All relevant study documentation and amendments were approved by the institutional review board of King Abdullah University Hospital (KAUH), Irbid, Jordan. The study was explained to patients when they met the inclusion criteria by their treating physician. All participants were assured that participation was voluntary and that withdrawal would not affect their treatment. Written informed consent was obtained from each eligible patient upon acceptance. Patient identity was kept anonymous.

### 2.4. Instrument

The Atrial Fibrillation Effect on Quality-of-life tool (AFEQT) was used. Systematic reviews in the literature showed that AFEQT was the highest-quality instrument among the six instruments used to assess QoL among AF patients [19,20]. This tool has two sections. The first determines the occurrence of atrial fibrillation, while the second section is a 7-point Likert scale used primarily to assess the level of QoL in patients with AF. The tool comprises 20 items asking about the magnitude (level) to which AF symptoms have impacted their QoL over the past four weeks. All items are rated from one (“Not at all bothered or I did not have this symptom”) to seven (“Extremely bothered”) [21]. The items are distributed across four domains: symptoms, daily activities, treatment concerns, and treatment satisfaction. The total score ranges from 0 to 100 and is derived from the summation of 18 items belonging to only three domains, including symptoms, daily activities, and treatment concerns. The higher score denotes better QoL [22]. The total score and domain score were calculated using the following equation: 100 − [(sum of severity for all questions answered − number of questions answered) × 100]/(total number of questions answered × 6) [23].

The symptoms domain (4 items) evaluates the extent to which AF symptoms (lightheadedness, palpitations, or dizziness) may have bothered patients over the past four weeks. The daily activities domain (8 items) estimates the extent of limitations patients experience when performing regular or vigorous physical activities or brisk walking [21]. The treatment concerns domain (6 items) assesses whether patients were worried or bothered by AF treatment therapy or exacerbated their medical condition. The treatment satisfaction domain (2 items) was excluded from the overall AFEQT summary score [21]. This tool is sufficiently reliable (Cronbach coefficient α = 0.90) [24] and valid in previous studies [10,13]. A QoL questionnaire was validated in this population before starting the study. In this study, Cronbach’s alpha was 0.92 for the total AFEQT and was 0.70, 0.87, 0.90, and 0.83 for the domains of symptoms, daily activities, treatment concerns, and treatment satisfaction, respectively. A Cronbach’s alpha ≥ 0.70 indicated adequate evidence of scale and subscales reliability [25].

### 2.5. Data Collection Procedure

After obtaining the patient’s approval during the clinic visit, the eligible patient was referred to meet the research assistant in a private room to complete the questionnaire. After that, the cardiac nurse provided the research assistant with the data relating to patients’ characteristics (weight, height, smoking, medications) and associated comorbidities (hypertension, valvular disease, systematic embolism, diabetes, chronic obstructive pulmonary diseases (COPD), dyslipidemia, strokes, and cancer). All research assistants underwent orientation about this study’s aims and the importance of consistency in data collection. Afterward, they participated in simulated environments about approaching patients, obtaining their consent, and working with cardiac nurses to collect relevant data using a standardized form.

### 2.6. Statistical Analysis

Data were analyzed using IBM SPSS Statistics, Version 29 (2023). Patient characteristics were compared across total AFEQT score quartiles using chi-square/Fisher’s exact test for categorical variables and Kruskal–Wallis test for continuous variables [10]. The chi-square and Fisher’s exact tests examine the relationship between categorical variables. In contrast, the Kruskal–Wallis test is a non-parametric method for comparing three or more independent groups on a continuous ordinal outcome variable [26]. Medians and interquartile ranges were calculated for continuous variables of patient characteristics. Further, mean, standard deviation (SD), median, and interquartile ranges were reported for the overall AFEQT score and AFEQT domains (symptoms, daily activities, treatment concerns, and treatment satisfaction). Concerning factors associated with QoL, a set of variables e.g., age, sex, BMI, history of diabetes mellitus, hypertension, dyslipidemia, stroke, COPD, CAD, and heart failure, type of atrial fibrillation (paroxysmal, persistent, long standing vs. reference: permanent), history of ablation, history of the occluded device, history of cardioversion, and history of using anticoagulants] were entered in a stepwise multiple regression. Stepwise multiple regression was used to determine the best-fit model that predicts the outcomes of the overall AFEQT score and its related domains (symptoms, daily activities, treatment concerns, and treatment satisfaction) and to identify factors that are independently associated with these outcomes.

## 3. Results

This study assessed 670 potential participants; 34 declined to participate, and 16 were excluded for not meeting the eligibility criteria (5 had a history of mental illness, 6 had cognitive disabilities, and 5 were illiterate), resulting in a final sample size of 620 participants. Half of the 620 patients were female. The median age of patients was 63 (IQR: 54.0–70.5) (Table 1).

Patients in the higher quartiles, indicating a better QoL, tended to be younger and were less likely to experience dyslipidemia, stroke, pulmonary hypertension, or other comorbidities (Table 1). Comorbidities associated with overall AFEQT score were CAD, cardiovascular, valve disease, and COPD or lung fibrosis (*p* ≤ 0.002). Ablation was uncommon (1.8%) but was associated with a better QoL (*p* = 0.001). AF symptoms were not related to the AFEQT score (*p* ≥ 0.093), although lower BMI and morbidity indices and higher LVEF were associated with a better QoL (*p* ≤ 0.002) (Table 1).

Over 90% of patients were currently experiencing AF, and patients with a better QoL tended to be less likely to be currently experiencing AF and more likely to have had their latest episode of AF more than a month ago (compared to less than a month ago) (Table 2). Prescriptions of medications were generally not clearly associated with QoL, although QoL improved as the proportion of patients prescribed any antiplatelet decreased (*p* = 0.013) (Table 2).

**Figure 1:**
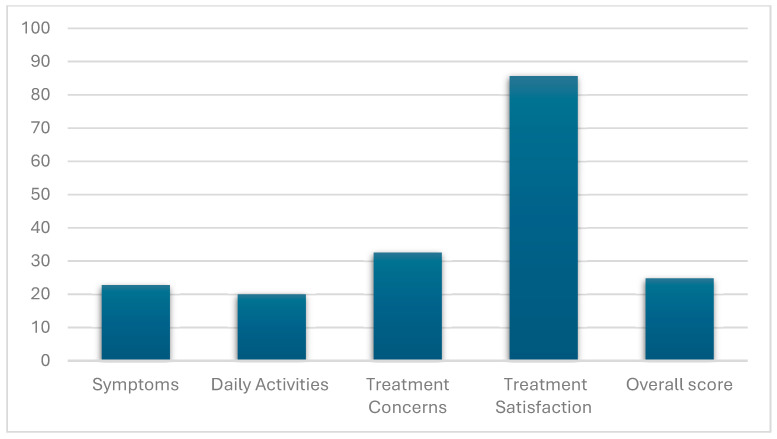
AFEQT average scores in AF patients (domains and overall score).

The overall AFEQT scores were positively skewed (median 21.3, IQR: 14.4–31.9). This pattern was reflected for the AFEQT sub-scales ‘Symptoms’ (median 20.8, IQR: 8.3–33.3), ‘Daily activities’ (median 16.7, IQR: 10.4–27.1) and ‘Treatment concerns’ (median 27.8, IQR: 19.4–41.7), whereas ‘Treatment satisfaction’ was negatively skewed (median 91.7, IQR: 83.3–91.7) (Table 3) (Figure 1).

Table 4 shows factors associated with the overall AFEQT score and domains of symptoms, daily activities, and treatment concerns. Age, BMI, dyslipidemia, heart failure, COPD, CAD, history of ablation, and the use of anticoagulants were significantly associated with the overall AFEQT score (R^2^ = 0.28). These eight factors could explain 28% of the overall QoL score among AF Jordanian patients. Patients with a history of ablation had a higher overall AFEQT score of 13.07 than those without a history of ablation (B = 13.07; 95%CI: 5.21, 20.92, *p* = 0.001). Meanwhile, patients with heart failure had a lower overall AFEQT score of 3.89 than those without heart failure (B = −3.89; 95%CI: −1.55, −6.23, *p* < 0.001). Age, BMI, dyslipidemia, heart failure, CAD, and the use of anticoagulants were significantly associated with symptom domain (R^2^ = 0.17). Age, BMI, dyslipidemia, heart failure, COPD, CAD, history of ablation, and the use of anticoagulants were independently and significantly associated with the daily activities domain (R^2^ = 0.22). Age, BMI, dyslipidemia, heart failure, COPD, CAD, history of ablation, and the use of anticoagulants were independently and significantly associated with the treatment concerns domain (R^2^ = 0.23). Regarding treatment satisfaction, no factor was found to be significantly related to this domain.

## 4. Discussion

The Jordan Atrial Fibrillation Registry Study shows three significant findings in this study: (1) low levels of QoL as exhibited by a low overall AFEQT score and its related domains of symptoms, daily activities, and treatment concerns; (2) patients in high quartiles of overall AFEQT scores were younger and with fewer number of comorbidities; (3) many patient characteristics, and diseases comorbidities were independently associated with the QoL in this population.

Our study shows participants had low mean and median scores overall and for most QoL domains. On the one hand, a similar pattern of low levels of QoL was reported by a few studies [27,28]. On the other hand, most other studies from different countries reported a higher QoL score (AFEQT) in patients with AF [10,29,30,31,32,33,34]. Reasons that could lead to such deviation in scores were challenging to point out, especially in the absence of earlier studies in Jordan that determine the QoL among this population. However, the low score of QoL once compared with other studies could be more complex and influenced by many factors, including sociodemographic characteristics (e.g., income), physical fitness, the mental health of participants, level of social support, knowledge about AF, and adherence with medical regimens and follow-up for AF and other comorbidities [10,12,13,34,35,36,37,38]. Further, the situation could be more impacted by COVID-19 infection and its related consequences of lockdown and interrupted medical services [39,40]. Further studies were required to shed light on the factors that could lead to such a low QoL in patients with AF in Jordan.

The classification of QoL in quartiles demonstrates a clear pattern of information. Compared to participants with low quartile scores, participants with higher quartile scores were found to be younger and with fewer comorbidities. Earlier studies reported similar findings [10,32].

Concerning factors that may be associated with QoL in AF patients, a set of modifiable and non-modifiable factors were identified in our study. Age, BMI, dyslipidemia, heart failure, COPD, CAD, history of ablation, and anticoagulant use were independently and significantly associated with the overall AFEQT score and the domains of daily activities and treatment concerns. The domain of symptoms has a similar pattern of association except for COPD and a history of ablation, which were found not to be significantly associated with this domain. Age increment was associated with lower overall QoL (B = −0.19; 95%CI: −0.29, −0.10, *p* = 0.001). Several studies reported similar findings [10,30,32]. Aging is well known to be associated with frailty and disease comorbidities, which correlates with lower QoL [41,42]. A higher BMI is another factor negatively associated with QoL (B = −0.82; 95%CI: −1.03, −0.62, < 0.001). This finding is in concordance with many other studies [10,31]. A study by Mitarni et al. (2022) reported that weight reduction and BMI significantly alleviated symptoms and improved QoL in AF patients [31]. In our study, gender was not associated with QoL in patients with AF. A contrary finding was reported by Randolph et al. (2016), which found that female patients had a poorer QoL than patients with AF. Such a difference in findings could be rationalized by the fact that gender could interact with other factors, such as age, which was higher in Randolph et al. (2016) than in our study. Thus, gender could be associated with more comorbidities and poorer QoL.

This study magnifies the benefit of cardiac ablation since it is significantly associated with higher QoL (B = 13.07; 95%CI: 5.21, 20.92, *p* = 0.001). Randomized clinical trials [43,44] and quasi-experimental [45], longitudinal [10,33,34], and cross-sectional observational studies reported a significant positive association between cardiac ablation and QoL among patients with AF [30,46]. In this study, the use of oral anticoagulants is another type of intervention found to be associated with higher overall scores for QoL and for the symptoms, daily activities, and treatment concerns domains. A study by Nakamaru et al. (2023) showed that AFEQT scores at baseline and one year after discontinuation did not differ significantly between patients who continued and those who discontinued the use of oral anticoagulants [47]. The current guidelines recommend prescribing oral anticoagulants to avoid stroke in patients with AF; however, prescribing oral anticoagulants requires strict adherence to follow-up; otherwise, the risk of bleeding could be increased [48]. Healthcare providers must balance the benefits and risks while using oral anticoagulants since they act as a double-edged sword that could improve or diminish the QoL in patients with AF.

Our study reported that a set of disease comorbidities (heart failure, COPD, CAD, and dyslipidemia) had an adverse effect on QoL. Heart failure, CAD, and COPD are known to decrease cardiac and respiratory capacity, which has great repercussions on other systems and the patient’s physical fitness. Earlier studies reported similar findings [10,33,49].

This study’s findings were found to be in concordance with many earlier studies; however, they differed in respect of reporting low levels of QoL in patients with AF. This study’s findings should be paramount since it is the first study on this population in Jordan. More studies are needed to understand this phenomenon. Further, QoL is one of the essential outcomes that healthcare providers need to pay closer attention to; it needs to be thoroughly monitored, and factors that could impede or improve QoL should be assessed to develop a tailored, individualized program for patients with AF. Healthcare providers should optimize medical management by ensuring that patients receive the most effective interventions, encouraging patients to adopt healthy lifestyles, enhancing their access to mental services, educating patients about their condition and treatment options, and enhancing their self-management strategies to alleviate AF-related symptoms and improve their QoL. Further, Jordan’s healthcare system should adopt policies and plans that enhance accessibility and quality of care for patients with AF.

## 5. Strengths and Limitations

This study is one of the first studies to shed light on factors associated with QoL among Jordanian patients with atrial fibrillation. Many explanatory factors, such as personal characteristics, disease comorbidities, AF characteristics, and AF-related interventions were explored in this study. This study enrolled patients from multicenter with diverse characteristics, which could help to generalize findings to a certain extent. Despite these strengths, there were many limitations. First, the design precludes causality. Second, this study depends on a self-reported AFEQT questionnaire, which makes data susceptible to recall bias. However, this measure is standardized, valid, and reliable. Third, the AFEQT score could be affected by the general health status of the participant. Fourth, despite a comprehensive coverage of explanatory variables, this study did not address other variables that could affect QoL (e.g., income, physical activity, social support, knowledge about AF, stress, depression, and anxiety). Fifth, clinical characteristics and diagnosis criteria were not assessed or evaluated by the research and were taken from clinics’ medical records. This reliance on existing records introduces the potential for bias, as the data collection and diagnosis methods may vary and are not standardized within the context of this study. Future research should consider limitations and cover more explanatory variables such as demographics, physical and mental status, and behavioral characteristics that may influence QoL in patients with AF.

## 6. Conclusions

This study demonstrates that AF Jordanian patients had low levels of QoL. Patients in higher quartiles for overall AFEQT score were younger, with fewer disease comorbidities and less experience of current AF episodes. Several modifiable and non-modifiable factors were associated with QoL in AF patients. Age, BMI, dyslipidemia, heart failure, COPD, CAD, history of ablation, and the use of oral anticoagulants were significantly associated with the overall AFEQT score. Healthcare providers should target these factors as indicators or interventions for which QoL is continuously monitored.

## Figures and Tables

**Table 1:** Patient characteristics across overall AFEQT score quartiles (N = 620).

	Overall (n = 620)	Quartile 1 (0–14.4) (n = 155)	Quartile 2 (14.5–21.3) (n = 157)	Quartile 3 (21.4–31.9) (n = 153)	Quartile 4 (32.0–100) (n = 155)	*p*
Age (y)	63.0 (54.0–70.5)	64.0 (55.0–70.0)	65.0 (55.0–72.0)	63.0 (54.0–71.0)	58.0 (53.0–67.0)	0.002
Female	312 (50.3)	77 (49.7)	75 (47.8)	86 (56.2)	74 (47.7)	0.397
Past medical history						
Hypertension	449 (72.4)	117 (75.5)	118 (75.2)	99 (64.7)	115 (74.2)	0.106
Diabetes	323 (52.1)	107 (69)	91 (58)	65 (42.5)	60 (38.7)	0.000
Smoker	105 (16.9)	30 (19.4)	28 (17.8)	19 (12.4)	28 (18.1)	0.377
Dyslipidemia	324 (52.3)	104 (67.1)	95 (60.5)	67 (43.8)	58 (37.4)	0.000
Stroke	78 (12.6)	26 (16.8)	21 (13.4)	21 (13.7)	10 (6.5)	0.045
Systemic embolization other than stroke	10 (1.6)	2 (1.3)	1 (0.6)	2 (1.3)	5 (3.2)	0.373 ^F^
Pulmonary Hypertension	157 (25.5)	55 (35.5)	34 (21.8)	31 (20.4)	37 (24.2)	0.009
Ablation	11 (1.8)	0 (0)	0 (0)	3 (2)	8 (5.2)	0.001 ^F^
Occluded device	2 (0.3)	0 (0)	1 (0.6)	1 (0.7)	0 (0)	0.621 ^F^
Electric cardioversion	38 (6.1)	8 (5.2)	10 (6.4)	6 (3.9)	14 (9)	0.280
Permanent pacemaker	9 (1.5)	1 (0.6)	2 (1.3)	4 (2.6)	2 (1.3)	0.517
Comorbidities	397 (64.0)	132 (85.2)	103 (65.6)	88 (57.5)	74 (47.7)	0.000
Comorbid conditions						
Sleep apnea	19 (3.1)	5 (3.2)	7 (4.5)	1 (0.7)	6 (3.9)	0.286
New stroke	45 (7.3)	16 (10.3)	11 (7)	9 (5.9)	9 (5.8)	0.378
Thyroid disease	62 (10)	15 (9.7)	14 (8.9)	12 (7.8)	21 (13.5)	0.364
Chronic kidney disease	46 (7.4)	16 (10.3)	14 (8.9)	4 (2.6)	12 (7.7)	0.056
Cancer	22 (3.5)	6 (3.9)	5 (3.2)	6 (3.9)	5 (3.2)	0.975
CAD	148 (23.9)	70 (45.2)	39 (24.8)	26 (17)	13 (8.4)	0.000
HF	184 (29.7)	75 (48.4)	43 (27.4)	36 (23.5)	30 (19.4)	0.000
Cardiovascular	325 (52.4)	113 (72.9)	83 (52.9)	78 (51)	51 (32.9)	0.000
Valve disease	84 (13.5)	15 (9.7)	22 (14)	34 (22.2)	13 (8.4)	0.002
DVT/PE	1 (0.2)	0 (0)	1 (0.6)	0 (0)	0 (0)	1.000 ^F^
WPW	2 (0.3)	0 (0)	2 (1.3)	0 (0)	0 (0)	0.249 ^F^
HOCM	1 (0.2)	0 (0)	0 (0)	1 (0.7)	0 (0)	0.247 ^F^
COPD or Lung fibrosis	45 (7.3)	25 (16.1)	13 (8.1)	4 (2.6)	3 (1.9)	0.000
AF symptoms						
Palpitations	297 (47.9)	74 (47.7)	71 (45.2)	78 (51)	74 (47.7)	0.793
Fatigue	149 (24)	31 (20)	42 (26.8)	38 (24.8)	38 (24.5)	0.554
Dizziness	81 (13.1)	14 (9)	23 (14.6)	25 (16.3)	19 (12.3)	0.313
SOB	227 (36.6)	53 (34.2)	59 (37.6)	61 (39.9)	54 (34.8)	0.714
Syncope	12 (1.9)	2 (1.3)	2 (1.3)	7 (4.6)	1 (0.6)	0.093 ^F^
Chest pain	5 (0.8)	2 (1.3)	0 (0)	1 (0.7)	2 (1.3)	0.555 ^F^
Asymptomatic	151 (24.4)	41 (26.5)	39 (24.8)	36 (23.5)	35 (22.6)	0.871
BMI	29.7 (25.9–33.2)	33.2 (30.0–36.7)	30.4 (26.0–33.1)	28.3 (25.4–31.2)	27.3 (24.8–31.1)	0.000
Left atrial size (cm)	4.2 (3.8–4.7)	4.2 (3.9–4.7)	4.3 (3.8–4.9)	4.0 (3.7–4.8)	4.2 (3.9–4.5)	0.428
LVEF (%)	55.0 (44.0–60.0)	50.0 (37.0–60.0)	55.0 (45.0–60.0)	55.0 (45.0–60.0)	57.0 (50.0–60.0)	0.002
Morbidity index	3.0 (2.0–4.0)	4.0 (3.0–5.0)	3.0 (2.0–4.0)	2.0 (1.0–3.0)	2.0 (1.0–3.0)	0.000

Values are presented as n (%) and median (interquartile range). CAD: coronary artery disease; HF: heart failure; DVT/PE: deep vein thrombosis/pulmonary embolism; WPW: Wolff–Parkinson–White syndrome; HOCM: hypertrophic cardiomyopathy; COPD: chronic obstructive pulmonary disease; SOB: shortness of breath; BMI: body mass index; LVEF: left ventricular ejection fraction. ^F^—Fisher’s exact test.

**Table 2:** Patients’ AF characteristics across overall AFEQT score quartiles (N = 620).

	Overall (n = 620)	Quartile 1 (0–14.4) (n = 155)	Quartile 2 (14.5–21.3) (n = 157)	Quartile 3 (21.4–31.9) (n = 153)	Quartile 4 (32.0–100) (n = 155)	*p*
AF Status						
Currently in AF	563 (90.8)	154 (99.4)	151 (96.2)	125 (81.7)	133 (85.8)	0.000
Latest episode more than a month ago	205 (33.3)	40 (26.1)	36 (22.9)	45 (29.4)	84 (54.9)	0.000
Left ventricular hypertrophy	214 (37.7)	54 (36)	52 (37.7)	53 (37.3)	55 (40.1)	0.91
Echo data available	593 (95.6)	151 (97.4)	145 (92.4)	150 (98.0)	147 (94.8)	0.080
Some echo finding	112 (18.2)	31 (20)	26 (16.7)	31 (20.3)	24 (51.7)	0.643
Valvular AF						
Non rheumatic or metallic valve	543 (87.7)	143 (92.3)	137 (87.3)	121 (79.1)	142 (92.2)	0.001
Moderate to severe mitral stenosis	36 (5.8)	6 (3.9)	7 (4.5)	19 (12.4)	4 (2.6)	
Prosthetic metal valve	40 (6.5)	6 (3.9)	13 (8.3)	13 (8.5)	8 (5.2)	
Medication/prescriptions						
Oral anticoagulant	485 (78.2)	107 (69)	128 (81.5)	129 (84.3)	121 (78.1)	0.007
Aspirin	273 (44)	78 (50.3)	69 (43.9)	70 (45.8)	56 (36.1)	0.086
Clopidogrel	71 (11.5)	21 (13.5)	19 (12.1)	13 (8.5)	18 (11.6)	0.56
Ticagrelor	8 (1.3)	2 (1.3)	3 (1.9)	3 (2)	0 (0)	0.385
Prasugrel	1 (0.2)	1 (0.6)	0 (0)	0 (0)	0 (0)	0.5 ^F^
Any antiplatelet	270 (43.5)	82 (52.9)	65 (41.4)	69 (45.1)	54 (34.8)	0.013
Two antiplatelets	41 (6.6)	9 (5.8)	14 (8.9)	7 (4.6)	11 (7.1)	0.457
Beta-Blocker	504 (81.3)	128 (82.6)	121 (77.1)	132 (86.3)	123 (79.4)	0.18
Amiodarone	130 (21)	32 (20.6)	36 (22.9)	37 (24.2)	25 (16.1)	0.318
Calcium channel blockers	61 (9.8)	12 (7.7)	15 (9.6)	17 (11.1)	17 (11)	0.522 ^F^
Digoxin	119 (19.2)	21 (13.5)	35 (22.3)	36 (23.5)	27 (17.4)	0.097
Flecainide, tambocor or encainide	15 (2.4)	4 (2.6)	3 (1.9)	2 (1.3)	6 (3.9)	0.520 ^F^
RAAS inhibitor (ACE/ARB/Entertsto)	215 (34.7)	51 (32.9)	53 (33.8)	52 (34)	59 (38.1)	0.779
Statin	214 (34.5)	58 (37.4)	56 (35.7)	51 (33.3)	49 (31.6)	0.719
Diuretic	223 (36)	58 (37.4)	59 (37.6)	58 (37.9)	48 (31)	0.522

Values are presented as n (%); ^F^—Fisher’s exact test.

**Table 3:** AFEQT scores: total and domains (N = 620).

AFEQT	Mean ± SD	Median (IQR)
Symptoms	22.73 ± 16.48	20.83 (8.33–33.33)
Daily Activities	19.95 ± 15.58	16.67 (10.42–27.08)
Treatment Concerns	32.56 ± 20.91	27.78 (19.44–41.67)
Treatment Satisfaction	85.66 ± 15.32	91.67 (83.33–91.67)
Overall score	24.77 ± 15.31	21.30 (14.4–31.90)

**Table 4:** Factors associated with AFEQT score (N = 620).

	B	SE	Beta	95% CI	*p*
Overall AFEQT score					
Age	−0.19	0.05	−0.15	−0.29, −0.10	0.001
BMI	−0.82	0.10	−0.29	−1.03, −0.62	<0.001
Dyslipidemia	−3.35	1.12	−0.11	−5.54, −1.16	0.003
Heart Failure	−3.89	1.19	−0.12	−6.23, −1.55	0.001
COPD	−5.34	2.09	−0.09	−9.44, −1.24	0.011
CAD	−7.03	1.27	−0.20	−9.53, −4.53	<0.001
History of Ablation	13.07	4.00	0.11	5.21, 20.92	0.001
Oral Anticoagulant	4.53	1.33	0.12	1.90, 7.16	0.001
Model summary: F = 29.34, *p* < 0.001, R^2^ = 0.28, Adjusted R^2^ = 0.27.					
Symptoms					
Age	−0.15	0.06	−0.10	−0.26, −0.04	0.008
BMI	−0.74	0.12	−0.24	−0.96, −0.51	<0.001
Dyslipidemia	−2.54	1.28	−0.08	−5.05, −0.02	0.048
Heart Failure	−4.18	1.37	−0.12	−6.86, −1.50	0.002
CAD	−7.26	1.46	−0.19	−10.13, −4.40	<0.001
Oral Anticoagulant	3.70	1.54	0.09	0.69, 6.72	0.016
Model summary: F = 21.40, *p* < 0.001, R^2^ = 0.17, Adjusted R^2^ = 0.17.					
Daily Activities					
Age	−0.23	0.05	−0.18	−0.33, −0.13	<0.001
BMI	−0.69	0.12	−0.23	−0.88, −0.46	<0.001
Dyslipidemia	−2.92	1.18	−0.09	−5.24, −0.61	0.014
Heart Failure	−3.88	1.26	−0.11	−6.35, −1.41	0.002
COPD	−4.98	2.21	−0.08	−9.31, −0.64	0.025
CAD	−6.35	1.34	−0.17	−8.94, −3.71	<0.001
History of Ablation	9.42	4.30	0.08	1.12, 17.73	0.026
Oral Anticoagulant	4.28	1.42	0.11	1.51, 7.06	0.003
Model summary: F = 21.54, *p* < 0.001, R^2^ = 0.22, Adjusted R^2^ = 0.21.					
Treatment Concerns					
Age	−0.17	0.07	−0.10	−0.31, −0.04	0.013
BMI	−1.01	0.16	−0.28	−1.37, −0.80	<0.001
Dyslipidemia	−4.56	1.58	−0.11	−7.65, −1.46	0.004
Heart Failure	−3.86	1.68	−0.08	−7.16, −0.55	0.022
COPD	−6.58	2.95	−0.08	−12.37, −0.78	0.026
CAD	−7.85	1.80	−0.16	−11.38, −4.32	<0.001
History of Ablation	21.17	5.65	0.13	10.01, 32.27	<0.001
Oral Anticoagulant	5.51	1.89	0.11	1.80, 9.22	0.004
Model summary: F = 22.32, *p* < 0.001, R^2^ = 0.23, Adjusted R^2^ = 0.22.					

## Data Availability

The data that support the findings of this study are available upon reasonable request.
